# Fine Mapping Identifies a New QTL for Brown Rice Rate in Rice (*Oryza Sativa* L.)

**DOI:** 10.1186/s12284-016-0076-7

**Published:** 2016-02-04

**Authors:** Deyong Ren, Yuchun Rao, Lichao Huang, Yujia Leng, Jiang Hu, Mei Lu, Guangheng Zhang, Li Zhu, Zhenyu Gao, Guojun Dong, Longbiao Guo, Qian Qian, Dali Zeng

**Affiliations:** State Key Lab for Rice Biology, China National Rice Research Institute, Hangzhou, 310006 P. R. China; College of Chemistry and Life Sciences, Zhejiang Normal University, Jinhua, 321004 P. R. China

**Keywords:** Brown rice ate, QTLs, Fine mapping, Candidate genes, Histocytological analysis

## Abstract

**Background:**

High yield and quality determine the commercial potential of rice variety. Brown rice rate (BRR) is a key factor ensuring grain yield and quality in rice. So far, there were few reports about the genes that directly controlled the BRR in rice.

Therefore, dissecting the genetic mechanism of the BRR genes can facilitate improving effective rice supply or edible grain yield.

**Results:**

A double haploid population derived from the cross between Taichung Native 1 (TN1) (an indica variety) and Chunjiang 06 (CJ06) (a japonica variety) was used to investigate the genetic basis of grain milling and appearance traits affecting the BRR. By using a constructed molecular linkage map, four quantitative trait loci (QTLs) for the BRR were detected on chromosomes 1, 8, 9, and 10, respectively. In addition, three QTLs for appearance traits, including grain weight and grain length/width ratio, were detected on chromosomes 6, 9 and 10, respectively. Chromosome segment substitution lines (CSSLs) were established at the *qBRR-10* locus. Finally, the *qBRR-10* was narrowed to a 39.5 kb region on chromosome 10. In this region, two candidate genes, *LOC_Os10g32124* and *LOC_Os10g32190*, showed significantly differential expression in TN1 and CSSL1-2 compared with CJ06. Histocytological analysis suggested that cell size and hull thickness may be important factors for the BRR.

**Conclusion:**

In the study, the *qBRR-10* affected the BRR and was finally located to a region between two markers, P13 and P14. Two candidate genes were selected based on the expression difference between two parents, which facilitated the further cloning of the *qBRR-10* gene and largely contributed to improve the grain yield and quality in rice*.*

**Electronic supplementary material:**

The online version of this article (doi:10.1186/s12284-016-0076-7) contains supplementary material, which is available to authorized users.

## Background

Rice is a major food staple for human. Currently, food security is becoming an ever more serious problem due to the pressure from continuous population growth and the increasing competition for arable land between food and energy crops (Gao et al., 2013). Therefore, it is urgent to secure grain production, which can be achieved by increasing grain yield and quality (Rosegrant and Cline, 2003). In most cases, the weight of brown rice is directly related to yield. Although rice hulls play a vital role in grain yield, they cannot serve as food for human. Therefore, BRR is an important trait in rice, which is also suggested by the milling quality effect (Tan et al., 2000; Aluko et al., 2004; Wan et al., 2008).

Based on molecular marker linkage maps and genomic sequencing, a large number of genes or quantitative trait loci (QTLs) related to yield traits have been successfully identified and applied in rice production. Dissection and characterization of these genes or QTLs have largely promoted the understanding of underlying mechanisms regulating yield traits in rice (Xing and Zhang, 2010; Guo and Ye, 2014), and have provided very helpful targets for improving grain yield and quality (Xing and Zhang, 2010; Wang et al., 2015b). *GRAIN SIZE 3* (*GS3*) encodes a putative transmembrane protein which has major impacts on grain length and weight as well as minor impacts on grain width and thickness in rice (Fan et al., 2006; Zhang et al., 2014). *GRAIN SIZE 5* (*GS5*) encodes a putative serine carboxypeptidase which controls grain size in rice by regulating grain width, filling and weight (Li et al., 2011). Grain length QTL (*qGL3*/*GL3.1*) encodes a putative protein phosphatase with Kelch-like repeat domain which can be used to significantly increase grain yield in rice (Qi et al., 2012; Zhang et al., 2014). *GRAIN WIDTH8* (*GW8/OsSPL16*) encodes a positive regulator of cell proliferation which can be used to promote cell division and grain filling (Wang et al., 2012). *GRAIN LENGTH7/GRAIN WIDTH7* (*GL7*/*GW7*) encodes a putative microtubule-binding protein which can regulate gain length and grain size diversity (Wang et al., 2015a; Wang et al., 2010). Therefore, function analysis of the genes related to seed traits is very important and can help to understand the genetic mechanisms regulating the BRR.

Generally, japonica varieties have higher BRRs than indica varieties. Wide variations of BRR are evident in rice germplasms, and most modern elite rice varieties show higher BRR than particular landraces (Luo and Yang 1998). Zhang and Peng (2012) reported that there was no significantly difference in the BRR, milled rice rate and head rice rate between reciprocal crosses. Although the factors determining the BRR have been studied for decades, no consensus has been reached. Chen et al. (1998) considered that BRR was mainly controlled by grain direct additive effects and maternal additive effect, and the latter is dominant. These results are in accordance with the additive-dominance model, and indicate that high BRR shows complete dominance or partial dominance over low BRR. However, cytoplasm effect is not significant. Besides, BRR is controlled by nuclear genes but not by maternal effects and cytoplasm effects. However, little attention is focused on the genetic basis of BRR in previous studies. It is reported that hybrid BRR, head rice rate, as well as the male parent and mid-parent values have significantly positive correlations (Tan et al., 2000; Luo et al., 2014). Using a double haploid (DH) population derived from the cross *O. sativa* × *O. glaberrima*, Aluko detected three QTLs for the BRR on chromosomes 1, 7, and 8, respectively (Aluko et al., 2004).

In the present study, a genetic analysis of QTLs determining BRR was carried out using a DH population derived from the cross between the indica rice cultivar Taichung Native 1 (TN1) and the japonica variety Chunjiang 06 (CJ06). Using the developed Chromosome segment substitution lines (CSSLs) population, *qBRR-10* was identified and finally narrowed down to a 39.5 kb region between two sequence-tagged site (STS) markers on chromosome 10. Two candidate genes in this region showed significant expression differences in TN1 and CSSL1-2 compared with CJ06. They might be responsible for the BRR. The genetic basis for other grain traits was also tested. The BRR related genes or QTLs identified in this study may be helpful for dissecting the genetic basis of grain yield and quality characteristics, which can facilitate the development of strategies to improve milling quality and grain yield.

## Results

### Phenotypic Variation of BRR in the DH Population

**Fig. 1:**
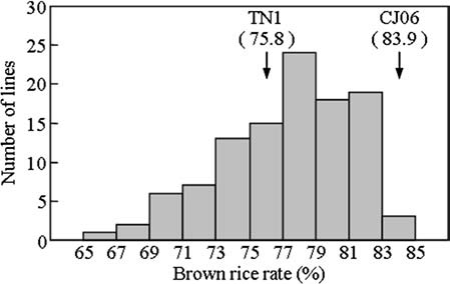
The distribution of brown rice rate in the DH population

The BRRs of the DH population and its parents were shown in Fig. 1. Significant differences in the BRR were found between two parents. The Parent CJ06 had a higher BRR reaching 83.9 %. However, the BRR of the parent TN1 was as low as 75.8 %. The BRR showed a continuous distribution from 66 % to 84 % among 120 DH lines, with an apparent peak at 78 %. Transgressive variation was also found in the DH population (Fig. 1).

### QTL Analysis for BRR

**Fig. 2:**
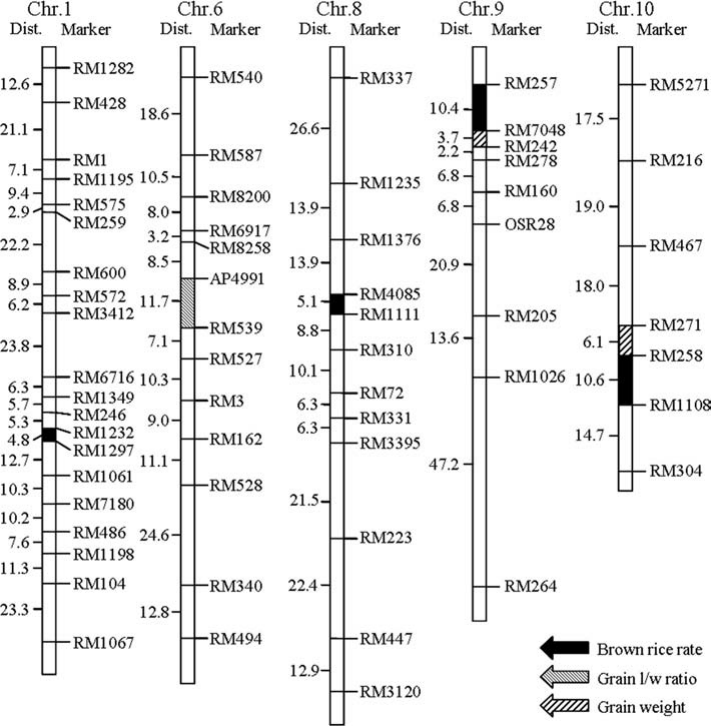
Locations of the QTLs for appearance and milling quality traits of rice grains. Only those portions of the linkage maps where QTLs were detected are shown

**Fig. 3:**
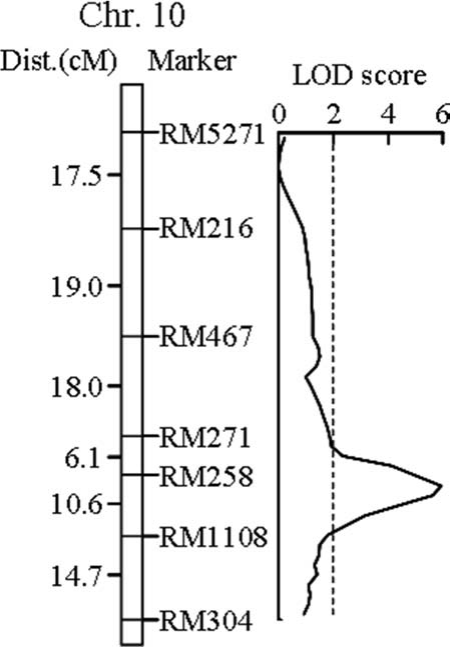
Molecular linkage map of rice chromosome 10 showing the location of *qBRR-10*. The genetic distance (Kosambi, centiMorgans) and marker name are shown on the left and right of the chromosome, respectively

The whole genome was scanned for detecting QTLs using Mapmaker/ QTL1.1B with a LOD threshold of 2.4. The analysis of the DH population identified four QTLs for the BRR located on chromosomes 1, 8, 9 and 10, respectively (Fig. 2, Additional file 1: Table S1). The QTL located in the interval RM258-RM1108 on chromosome 10 showed the largest effect on the BRR trait with an LOD score of 5.95, and accounted for 23.1 % of the phenotypic variances (Fig. 3). The effects of the other three QTLs were much smaller. The alleles in all of the four QTLs from CJ06 contributed to the increased BRR.

### Correlation Analysis

Correlation analysis between the BRR and other seed traits was performed using the software SAS 8.0. Other seed traits included grain length, grain width, grain length/width ratio, grain thickness, grain weight, brown rice length, brown rice width, brown rice length/width ratio, brown rice thickness and brown rice weight. The distribution of these traits in the DH population was shown in Additional file 1: Figure S1. Our results suggest that BRR is positively correlated with the brown rice weight, brown rice width, and has no relationship with other seed traits. Moreover, there were extremely significant relationships between the BRR and brown rice weight, grain thickness and brown rice thickness (Table 1), and our measurement data also supported it (Additional file 1: Figure S2). Meanwhile, the QTLs for other seed traits were also investigated using the same linkage map. Three QTLs were detected: one for grain length/width ratio and two for grain weight, and the intervals of two grain weight QTLs were near from those of BRR QTLs (Fig. 2, Additional file 1: Table S2).

**Table 1:** Correlation analysis between brown rice rate and other seed traits

	Grain length	Grain width	Grain l/w ratio	Brown rice length	Brown rice width	Brown rice l/w ratio	Grain weight	Brown rice weight	Brown rice rate	Grain thickness
Grain width	0.1263									
Grain l/w ratio	0.6626^b^	-0.6546^b^								
Brown rice length	0.6721^b^	-0.00002	0.5126^b^							
Brown rice width	-0.1103	0.616^b^	-0.5514^b^	-0.0441						
Brown rice l/w ratio	0.5276^b^	-0.4197^b^	0.7232^b^	0.7219^b^	-0.7194^b^					
Grain weight	0.3454^b^	0.5463^b^	-0.1561	0.4173^b^	0.66^b^	-0.1679				
Brown rice weight	0.3066^b^	0.5213^b^	-0.1666	0.4116^b^	0.6512^b^	-0.165	0.9643^b^			
Brown rice rate	-0.018	0.1005	-0.0911	0.1201	0.1928^a^	-0.0485	0.1739	0.425^b^		
Grain thickness	-0.0456	0.5109^b^	-0.3991^b^	-0.0804	0.6612^b^	-0.4981^b^	0.7434^b^	0.7542^b^	0.3175^b^	
Brown rice thickness	0.0032	0.4579^b^	-0.3551^b^	0.0035	0.6296^b^	-0.4273^b^	0.772^b^	0.7914^b^	0.3075^b^	0.8728^b^

^a^, ^b^significant at the level of 5 % and 1 %, respectively

### Substitution Mapping of *qBRR-10*

**Fig. 4:**
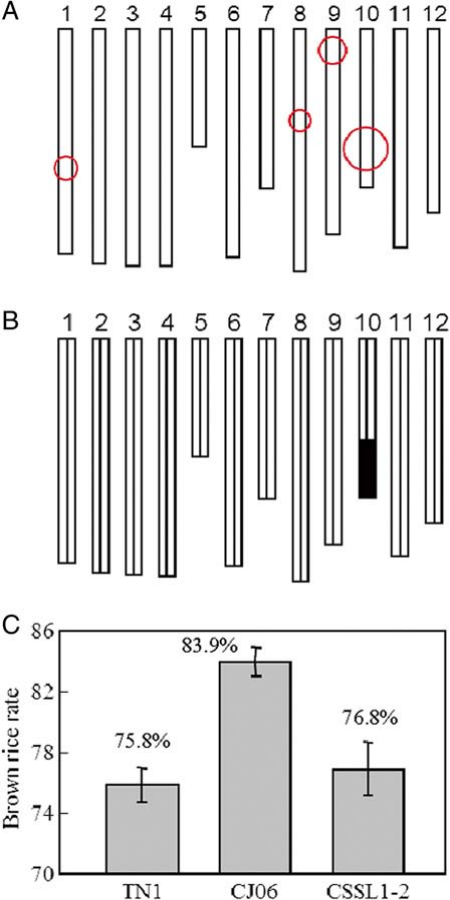
The development of CSSL. **a** QTL analysis for brown rice rate in DH population. Circle centers indicate positions of QTLs on the rice chromosomes. Circle sizes indicate contributions to explained variation for brown rice rate. **b** Graphical genotype of CSSL1-2 (a substitution line of chromosome 10. Black bar indicates the genome fragment from TN1; the other parts were from CJ06. **c** The brown rice rate for CJ06, TN1 and CSSL1-2

Three lines from the DH population were selected to backcross with CJ06 for 4 rounds. According to the QTL analysis and a previous genetic linkage map, the SSR markers RM271 and RM304 were used in marker-assisted selection for segregating the progenies carrying *qBBR-10* during every generation of backcross. After 4 rounds of backcrosses with CJ06, the BC_4_F_1_ and BC_4_F_2_ generations were scanned with a set of 71 SSR markers, which were uniformly distributed on a previous linkage map (Additional file 1: Table S4). The plant CSSL1-2 containing a small amount of TN1 DNA was selected. It carried a homozygous introgression across the entire *qBRR-10* region, but without any introgression across *qBRR-1, qBRR-8* and *qBRR-9* regions on chromosomes 1, 8, and 9, respectively (Fig. 4a and b). The BRR of CSSL1-2 was significantly lower than that of its recurrent parent CJ06, but was much more similar to that of TN1 (Fig. 4c).

### Fine Mapping of *qBRR-10* and Analyzing Candidate Genes

**Fig. 5:**
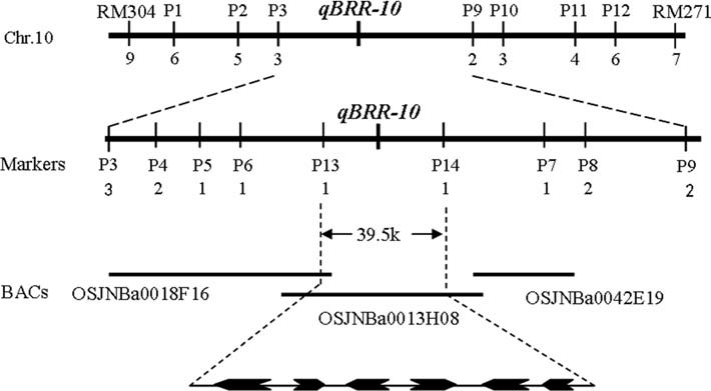
Fine mapping of *qBRR-10*

**Fig. 6:**
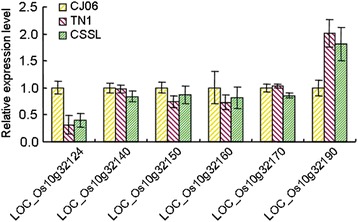
The qPCR analysis of predicted genes in CJ06, TN1 and CSSL1-2

The simple sequence repeat (SSR) markers RM271 and RM304 across the *qBRR-10* target region were used to determine the recombination breakpoints in segregating progenies derived from the cross between CSSL1-2 and CJ06. A total of 235 selected plants from 910 BC_4_F_2_ progenies were cultivated in the paddies to gain enough seeds for the BRR evaluation and further study. These plants could be divided into two subgroups based on the detected BRR values. One group was with higher BRR, while the other group was with lower BRR. A total of 41 plants with extremely low BRRs (*BRR* < =72.6) were selected for fine mapping and the use of heterozygous plants or confounding plants was avoided. Then 9 and 7 recombination events between the marker and *qBRR-10* were identified based on analyses using RM271 and RM304, respectively. The STS markers P1, P2 and P3 revealed 6, 5 and 3 recombinants, respectively; the markers P11, P10 and P9 indicated 4, 3 and 2 recombinants, respectively (Fig. 5). Therefore, the *qBRR-10* locus was finally located to a region between two STS markers, P13 and P14 on the BAC OSJNBa0013H08 (Fig. 5 and Additional file 1: Table S3). By comparison, it was found that the interval was 39.5 Kb. The target region contains 6 predicted genes (*LOC_Os10g32124*, *LOC_Os10g32140*, *LOC_Os10g321250*, *LOC_Os10g32160*, *LOC_Os10g32170* and *LOC_Os10g32190*) based on Rice Genome Annotation Project (http://rice.plantbiology.msu.edu/cgi-bin/gbrowse/rice/). We performed a quantitative reverse transcription-PCR (qPCR) analysis to determine the candidate genes, and the analysis results showed that no significant difference was detected in *LOC_Os10g32140*, *LOC_Os10g32150*, *LOC_Os10g32160* and *LOC_Os10g32170* among CJ06, TN1 and CSSL1-2 (Fig. 6 and Additional file 1: Table S3). On the contrary, *LOC_Os10g32124* and *LOC_Os10g32190* showed significantly different expression levels in TN1 and CSSL1-2 compared with CJ06 (Fig. 6 and Additional file 1: Table S3). Therefore, *LOC_Os10g32124* and *LOC_Os10g32190* were likely to be the candidate genes for *qBRR-10*.

### Histocytological Analysis

**Fig. 7:**
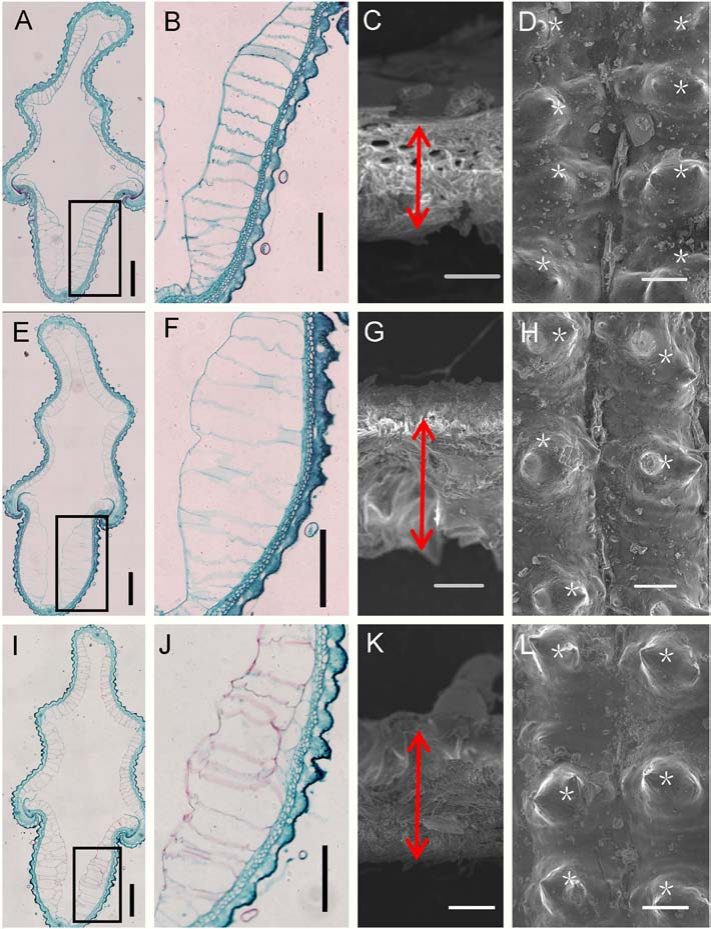
Histocytological analysis of hulls in CJ06, TN1 and CSSL1-2. **a** paraffin section of hull in CJ06. **b** partial magnification of black box region in **a**. **c** hull thickness in CJ06. **d** Epidermal surface of hull in CJ06. **e** paraffin section of hull in TN1. **f** partial magnification of black box region in **e**. **g** hull thickness in TN1. **h** Epidermal surface of hull in TN1. **i** paraffin section of hull in CSSL1-2. **j** partial magnification of black box region in **i**. **k**, hull thickness in CSSL1-2. **l** Epidermal surface of hull in CSSL1-2. Bars = 200 μm in **a**, **b**, **e**, **f**, **i**, **j** and 20 μm in **c**, **d**, **g**, **h**, **k**, **l**

Because the hull percentage is negatively correlated with milling quality (Jongkaewwattana and Geng 2001), we examined the hulls of CJ06, TN1 and CSSL1-2 using paraffin section and scanning electron microscopy (SEM) to reveal the impacts of hull effect on the BRR. Hulls are comprised of four layers: silicified cells, fibrous sclerenchyma, spongy parenchymatous cells and nonsilicified cells from outside to inside (Ren et al., 2013). The hulls of TN1 and CSSL1-2 showed larger nonsilicified and silicified cells compared to CJ06 (Fig. 7a, b, e, f, I and j). SEM analysis showed that TN1 and CSSL1-2 hulls were thicker than those of CJ06 (Fig. 7c, g and k, and Additional file 1: Figure S2), which was consistent with the results of paraffin sections. These results suggest that *qBRR-10* is involved in regulating hull development, and CJ06 variety has higher BRR and provides more grain productivity since the percentage of hull is negatively correlated with the BRR. A higher cell density (i.e., protrusions ) was also observed in the outer surface of CJ06 hulls than those of TN1 and CSSL1-2, which was consistent with the observation that the grains of CJ06 were longer than those of TN1 (Figs. 7d, h and l). Hulls control the vital exchanges of gas, water and nutrients with environment, as well as affect seed development and grain filling (Javelle et al., 2011). These results provide valuable background information on the organization of hull tissues, which facilitate the understanding of molecular mechanisms determining BRR.

## Discussion

Rice grain consists of an inner brown rice (edible part) and a hull (inedible part) reciprocally interlocked by the lemma and palea (Yang, 2005). Converting photosynthetic products to people edible part or brown rice as far as possible was one of the effective methods to increase the “output” of rice. Therefore, increasing the grain the BRR or decreasing the hull percentage played a vital role in improvement of the actual grain yield. However, BRR was related to many seed traits, including grain length, grain width, grain weight, brown rice weight and hull weight etc. Generally, grain length, grain width, grain length/width ratio and the translucency of endosperm directly determined grain appearance and quality (Tan et al., 2000; Koutroubas et al., 2004; Wang et al., 2015a; Xing and Zhang, 2005; Li et al., 2014). Currently, a lot of QTLs and genes related to these traits had been cloned, such as *GRAIN WIDTH2* (*GW2*), *GS3*, *qGL3*/*GL3.1*, *GW8/OsSPL16*, *SMALL AND ROUND SEED 3* (*SRS3*), *POSITIVE REGULATOR OF GRAIN LENGTH 1* (*PGL1*) and *GL7/GW7* (Fan et al., 2006; Song et al., 2007, Kanako et al., 2010, Heang and Sassa 2012, Wang et al., 2012, Zhang et al., 2014, Wang et al., 2012; Wang et al., 2015b)*.* These QTLs and genes controlled the brown rice size and weight by regulation of cell proliferation and expansion of the hull, and further promoted the application of molecular breeding technology. These results had significant implications for the improvement of effective rice supply or edible grain and that the hull may be involved in regulation of brown rice yield or BRR. So far, there were few reports about the genes that directly controlled the BRR in rice. Therefore, dissecting the genetic mechanism of the BRR genes can facilitate to improve effective rice supply or edible grain yield.

In this study, the fine mapped *qBRR-10*, a major potential QTL controlling the BRR, was reported. The major QTL for BRR detected in the present study on chromosome 10 was different from the results of previous studies (Aluko et al., 2004; Kepiro et al., 2008). However, there was a QTL locus for rice bran on chromosome 10 (Aluko et al., 2004), which was located in the same region with the *qBRR-10*. Perhaps the major QTL on chromosome 10 controlled both BRR and rice bran. The other three BRR loci including the *qBRR-1*, *qBRR-8* and *qBRR-9* had minor effects. Finally, we fine mapped the *qBRR-10* gene between STS markers P13 and P14 on the long arm of chromosome 10 with a 39.5 kb physical distance in Nipponbare. In this region, there were six open reading frames and no known genes or QTLs were reported, so the *qBRR-10* gene was considered as a novel gene. Two candidate genes, *LOC_Os10g32124* and *LOC_Os10g32190*, encoding proteins with unknown functions, were identified based on their differences in gene transcription level. Thus more work, such as complementary test, was needed to examine whether *LOC_Os10g32124* or *LOC_Os10g32190* was the target gene for the *qBRR-10*.

Hulls, main parts of grains, affected grain size and BRR which were the main factors affecting the actual grain yield (Yang, 2009; Song et al., 2007; Xing and Zhang, 2010; Luo et al., 2014; Song et al., 2015). *GW2*, a major grain width and weight QTL, increased grain width and weight by enlarging hulls. The NIL (*GW2*) hull showed more cells than that of *FAZ1*, and resulted in a higher brown rice yield and BRR (Song et al., 2007). *ABNORMAL FLOWER AND DWARF1* (*AFD1*) determined plant height, floral development and grain yield. The thicker hulls in the *afd1* mutant caused reduced brown rice yield than that of the wild type. But, no differences were observed in the brown rice size and weight between the wild type and *afd1* mutant when parts of both hulls were removed (Ren et al., 2015). Meantime, our data also showed that brown rice of CJ06 was thicker than that of TN1 and CSSL1-2 (Additional file 1: Figure S2). These findings suggested that the rice hull and brown rice thickness may be involved in regulation of brown rice yield and BRR. In the study, paraffin sections and SEM analysis revealed that the TN1 hulls had larger nonsilicified and silicified cells, and thicker layers. The results indicated that cell size and hull layers may be important factors affecting the BRR. It provides very valuable information for cloning and functional analysis of the *qBRR-10* gene. Moreover, three more traits were detected in the present study, i.e., one for brown grain length/width ratio (*qGL/W-6*), and two for grain weight (*qGW-9* and *qGW-10*). The QTL on chromosome 6 had significant impact on grain length/width ratio, which had not been reported before. Thus, these QTLs identified in this study are very useful for studying the BRR and grain weight. The identification of *qBRR-10*, *qGL/W-6*, *qGW-9*, and *qGW-10* will receive much attention because of their importance on improving milling quality and rice yield.

In rice production, many factors can affect the BRR, such as processing equipments and environmental conditions. The content of inorganic elements can also affect the BRR. The study performed by Juliano and Villarea (1993)) suggests that the grains containing more minerals have higher brittleness, thus are more easily processed and lead to a lower BRR. The results from Zhang also showed that high iron and zinc contents caused a lower BRR in rice (Zhang and Peng, 1997). Therefore, more work is needed to determine the target gene of the *qBRR-10* and understand the genetic mechanisms controlling the BRR, which will provide a new opportunity for increasing the BRR and thus further improving grain yield and quality.

## Conclusion

In this study, using the DH population, 4 QTLs were detected in Hangzhou and Hainan to control the BRR. With the BC_4_F_2_ progenies derived from the cross between CSSL1-2 and CJ06, a new major QTL (*qBRR-10*) for the BRR, was fine mapped within 39.5 kb physical interval on chromosome 10. Two candidate genes were finally selected based on the difference in the transcriptional expression. The cloning and genetic mechanism study of the *qBRR-10* will facilitate increasing the BRR and further improving rice yield and milling quality.

## Methods

### DH Population

We constructed the DH population to detect BRR related QTLs in our lab (Ma et al. 2009a). The indica cultivar ‘TN1’ and the japonica cultivar ‘CJ06’ of rice (*Oryza sativa* L.) were used as parents to generate hybrids. TN1 had a lower BRR, while CJ06 was with higher BRR. The anthers from F_1_ plants were collected and cultured on the inducing medium SK3. After natural doubling or treatment with colchicines, DH plants were obtained, and have been successfully used for studying whitebacked planthopper resistances and ligule length (Sogawa et al., 2005; Sogawa et al., 2004; Zeng et al., 1997; Su et al., 2011). The DH population and its parents were transplanted in a density of 20 cm × 20 cm at rice growing season in the experimental farms at China National Rice Research Institute, Hangzhou, or in Lingshui County, Hainan Province. Each DH line was planted in six rows, and each row contained six plants in the year 2010 and 2011.

### The Development of CSSLs

To obtain CSSLs, the plants carrying TN1 genotype at the flanking region of *qBRR-10* were selected to backcross with CJ06 for 4 rounds. The SSR markers RM304 and RM271 were simultaneously used to identify the plants containing TN1 genotype in backcross lines. A set of 71 SSR markers (Additional file 1: Table S4) uniformly distributed on a previous linkage map (Sogawa et al., 2005; Sogawa et al., 2004; Zeng et al., 2009) were used to select the individual plants containing the least TN1 DNA in BC_4_F_1_ lines. CSSL1-2 was selected to fine map the *qBRR-10* genes. It contained a small amount of TN1 DNA in its genetic background, and carried a homozygous introgression across the entire *qBRR-10* region and without any introgression across *qBRR-1*, *qBRR-8* and *qBRR-9* regions on chromosomes 1, 8, and 9 respectively.

### DNA Extraction and PCR Analysis

Total DNA was extracted from fresh rice leaves via the CTAB method described by Murray and Thompson (1980) with slight modifications. PCR was performed in 20 μL reaction mixture containing 0.2 mM of each primer, 200 mM dNTP mix, 50 mM KCl, 10 mM TRIS-HCl, pH 8.3, 1.5 mM MgCl_2_, 0.1 % Triton X-100 and 1 unit of Taq polymerase. The PCR protocol was: initial denaturation at 94 °C for 5 min, followed by 35 cycles performed at 94 °C for 1 m, 55 °C for 45 s and 72 °C for 50 s, and then a final extension at 72 °C for 10 m. The PCR reaction was performed in PTC-225 tetrad (MJ Research, Watertown, MA) (Su et al., 2011). The PCR products were separated via electrophoresis on a 4.0-5.0 % (w/v) agarose gel and stained with nucleic acid dye.

### Marker Development

Primers were designed around *qBRR-10* on chromosome 10 to distinguish CJ06 and TN1 (Additional file 1: Table S3). The STS markers were developed based on the sequence differences between the indica var. 93-11 and the japonica var. Nipponbare (http://www.ncbi.nlm.nih.gov) (Additional file 1: Table S3) (Su et al., 2011). This strategy was helpful because of the close genetic relationship between CJ06 and Nipponbare and the relative sequence similarity between TN1 and 93-11. The sequences were aligned using the SeqMan program of DNA star (Gene-Codes). Meanwhile, insertions and deletions were identified. Primers flanking the indels were designed using the software Primer Premier 5.0 and tested on the parents.

### Grain Phenotyping

Rice grains were harvested and stored at room temperature for at least 3 months before processing. For each rice line, the hulls of 50 g rough grains were removed by a huller (manufactured by Jiading Food and Oil Machinery Factory, Shanghai, China) according to the National Standards NY 147-88. The whole and broken kernels obtained after milling were added to define the total milling yield. BRR was calculated with dividing total grain weight by brown rice weight.

The quality of appearance is determined by grain length, width, width/length ratio, size and shape, and translucency of endosperm (Juliano and Villareal., 1993; Unnevehr et al., 2006). Several appearance-related traits were also used to test whether they have any relationship with BRR. The length and width of 10 fully formed grains from each DH line were measured using a vernier caliper. The length/width ratio of the grains was calculated with dividing grain width by grain length. It reflects the shape of grains. Ten randomly selected unbroken brown rice grains of each DH line were lined up to measure grain length, width, and length/width ratio in the same way as described above. The thickness of 10 grains and 10 brown rice grains in each line were also measured using a vernier caliper.

### Data Analysis and QTL Mapping

The linkage map used in this study was established by the China National Rice Research Institute (Zeng et al., 2009). Recently, this linkage map has been widely used in QTL mapping (Ma et al. 2009b; Zeng et al., 2009). Analysis of variance for all phenotypic characters was performed using the JMP statistical package, version 7.0 for Windows (SAS Institute Inc., Cary, NC) (Su et al., 2011). QTL mapping for BRR was conducted using Mapmaker/QTL 1.1. The presence of QTL was claimed when LOD score was larger than 2.4. The genetic variances explained by each QTL and QTL additive effect were calculated. Moreover, the discovered QTLs were named according to the standard nomenclature, as suggested by McCouch et al. (1997).

### Paraffin Sections and Microscopic Analysis

The hulls of CJ06 and TN1 were collected at heading stage and fixed overnight at 4 °C in the solution containing 50 % ethanol, 0.9 M glacial acetic acid and 3.7 % formaldehyde, then dehydrated by a graded ethanol series, infiltrated with xylene, and embedded in paraffin (Sigma) (Ren et al. 2013). The 8 um-thick sections were transferred onto poly-L-Lys-coated glass slides, deparaffinized in xylene, and dehydrated through an ethanol series. The sections were sequentially stained with 1 % safranine (Amresco) and 1 % Fast Green (Amresco), then dehydrated through an ethanol series, infiltrated with xylene, and finally mounted beneath a coverslip (Ren et al., 2013). Light microscopy was performed using a Nikon SMZ1500 microscope. The samples were also examined using a Hitachi S-3400 scanning electron microscope.

### RNA Isolation and Qpcr Analysis

Total RNA was isolated using the RNeasy Plant Mini Kit from Watson. The first strand of complementary DNA was synthesized from 2 mg total RNA using oligo(dT)18 primers in a 25 mL reaction mixture from the SuperScript III Reverse Transcriptase Kit (Invitrogen). Reverse-transcribed RNA (0.5 mL) was used as PCR template for gene-specific primers (Additional file 1: Table S3) (Ren et al., 2013). The qPCR analysis was performed using SYBR Supermix Kit (Bio-Rad) in ABI Prism 7000 Sequence Detection System. At least three replicates were performed, and the mean expression level for each gene was calculated.

## Additional file

Additional file 1: Figure S1. The distribution of other seed traits in the DH population. **Figure S2.** The thickness of hull and brown rice in CJ06, TN1 and CSSL1-2. **Table S1.** QTL identified for brown rice rate in the DH population. **Table S2.** QTL identified for other related traits in the DH population. **Table S3.** Primers used in the study. **Table S4.** SSR markers selected to identify the CSSLs. (DOC 171 kb)

## Abbreviations

BRR: Brown rice rate
QTLs: Quantitative trait loci
CSSLs: Chromosome segment substitution lines
DH: Double haploid
STS: Sequence-tagged site
SSR: Simple sequence repeat
SEM: Scanning electron microscopy
