# Supplementary material for: Fine Mapping Identifies a New QTL for Brown Rice Rate in Rice (Oryza Sativa L.)
Source: Rice (N Y). 2016 Feb 4;9:4. doi: 10.1186/s12284-016-0076-7 (PMC4742455; doi:10.1186/s12284-016-0076-7)
Supplement: Additional file 1: Figure S1. — The distribution of other seed traits in the DH population. Figure S2. The thickness of hull and brown rice in CJ06, TN1 and CSSL1-2. Table S1. QTL identified for brown rice rate in the DH population. Table S2. QTL identified for other related traits in the DH population. Table S3. Primers used in the study. Table S4. SSR markers selected to identify the CSSLs. (DOC 171 kb) [file 12284_2016_76_MOESM1_ESM.doc]

**ADDITIONAL FILE FIGURE AND TABLE**

**Figure S1.** The distribution of other seed traits in the DH population


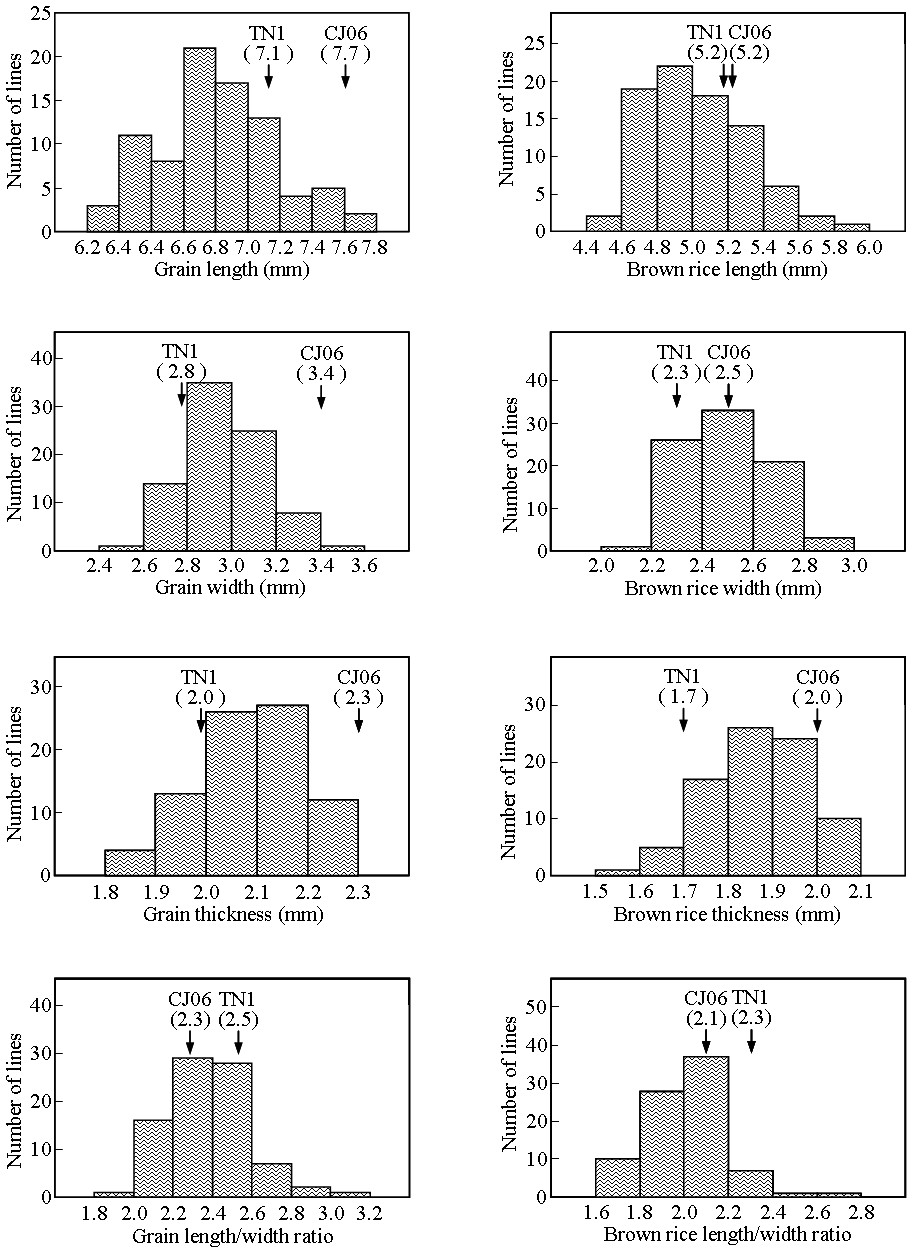


**Figure S2.** The thickness of hull and brown rice in CJ06, TN1 and CSSL1-2.

**Significant difference at P<0.01 compared with the CJ06.

**Table S1. QTL identified for brown rice rate in the DH population**

| Chromosome | Interval | LOD score | Variance explained | Additive effect |
| --- | --- | --- | --- | --- |
| 1 | RM1232-RM1297 | 2.73 | 8.00% | 2.895 |
| 8 | RM4085-RM1111 | 2.46 | 7.70% | -2.861 |
| 9 | RM257-RM7048 | 3.13 | 9.90% | -3.304 |
| 10 | RM258-RM1108 | 5.95 | 23.10% | -5.719 |

**Table S2.** QTL identified for other related traits in the DH population

| traits | critical_value | interval | chr. | F |
| --- | --- | --- | --- | --- |
| grain length | 11.81 | RM514-RM570 | 3 | 8.12 |
| grain width | 11.34 | RM154-RM3188 | 2 | 8.21 |
| grain length/width ratio | 11.17 | AP4991-RM539 | 6 | 17.37 |
| brown rice length | 11.41 | RM450-RM5472 | 2 | 7.22 |
| brown rice width | 11.88 | RM7-RM251 | 3 | 10.99 |
| brown rice length/width ratio | 11.44 | RM6917-RM8258 | 6 | 8.79 |
| grain thickness | 11.58 | RM7-RM251 | 3 | 8.2 |
| brown rice thickness | 12.08 | RM7-RM251 | 3 | 6.85 |
| grain weight | 10.69 | RM7018-RM242 | 9 | 11.25 |
| grain weight | 10.69 | RM271-RM258 | 10 | 11.52 |
| brown rice weight | 8.57 | RM216-RM467 | 10 | 6.2 |
| brown rice rate | 10.98 | RM271-RM258 | 10 | 17.62 |

**Table S3.** Primers used in the study.

| Purpose | Primer name | Sequence |
| --- | --- | --- |
| Mapping | P1-F | AGTGGTCGGTAATCAGCACTC |
|  | P1-R | GAATGGTTAAGTCATCAGTGCC |
|  | P2-F | GGAGGAGGTGTCCAAGG |
|  | P2-R | GCCATTCACATCACACTAACACATA |
|  | P3-F | GGATGGACGCTAACTCTAA |
|  | P3-R | TCTTTCTGTATTTTGTGGGA |
|  | P4-F | TAGAGTCTCCCATCCCAGTA |
|  | P4-R | CCTTGAGGAGCTACCATG |
|  | P5*-*F | GCCCAGAACACCAACTCCAGCAC |
|  | P5*-*R | CACTTGGGACGACGCCTGCTG |
|  | P6*-*F | TTTGGGTTCTGACTTCTG |
|  | P6*-*R | AGACCTTCCTTTTGAGC |
|  | P7*-*F | CTACACGCGCAAACTCTGTC |
|  | P7*-*R | ATGAAGGTCTAGGCTGCACC |
|  | P8*-*F | CAGCCCACCTTCGTCA |
|  | P8*-*R | CCCCCCAACACAAATGCCTAAAACA |
|  | P9*-*F | TTCATCCCCAAGCCCAAGG |
|  | P9*-*R | AAGAATCATCCATCCGGTCATC |
|  | P10*-*F | CTCTTTTCCCCTCTATCTCACTTCCTC |
|  | P10*-*R | CAACATCACCGACGCCACCG |
|  | P11*-*F  P11*-*R  P12F | GCGTTGCCAGAAAATGGT  CCTCCAAGCAGAATTGCC  ACCCTCGCGAATTTTTAAGC |
|  | P12*-*R | TGTCAGTGCTCGGTTTTGTC |
|  | P13*-*F | ATTTTGTAGCCATGTGCT |
|  | P13*-*R | TTATCTTGCTGTCATTGGA |
|  | P14*-*F | AAATGGGCGGTGGAACATG |
|  | P14*-*R | GACAGCAACAAGGCAAGGGA |
| qRT-PCR | *LOC_Os10g32124*-F | CCGATCCCGTTAGTGAGAATGA |
|  | *LOC_Os10g32124*-R | CACAAATTGGATGGAGGAATCG |
|  | *LOC_Os10g32140*-F | ACTTGAACTTCCTGATCGCCG |
|  | *LOC_Os10g32140*-R | GGAGGACGGCTCCTTCCTG |
|  | *LOC_Os10g32150*-F | CCACAGGGGTCTCCACGG |
|  | *LOC_Os10g32150*-R | GTAGACCACCGAGCCAGCC |
|  | *LOC_Os10g32160*-F | GTCACGATGCTGCTGCGC |
|  | *LOC_Os10g32160*-R | GAATCGGAGAACGACGGCC |
|  | *LOC_Os10g32170*-F | GCTACGACTCCGGCGTGAT |
|  | *LOC_Os10g32170*-R | GCGAATGGCCAGAGAACGA |
|  | *LOC_Os10g32190*-F | GCAGCTACAGGTTCCCACGA |
|  | *LOC_Os10g32190*-R | CCTACCTTGAAAGGGTTGACAGC |

**Table S4. SSR markers selected to identify the CSSLs.**

| Chromosome | SSR markers |
| --- | --- |
| Chr.1 | RM1282, RM5302, RM259, RM572, RM488, RM5461, RM1061, RM1067 |
| Chr.2 | RM3188, RM521, RM341, RM263, RM5472, RM535 |
| Chr.3 | RM489, RM5480, RM7, RM282, RM2334, RM3919, RM520, RM85 |
| Chr.4 | RM3471, RM548, RM3735, RM3276, RM1113 |
| Chr.5 | RM440, RM5642, RM6972, RM31 |
| Chr.6 | RM8200, RM8258, RM6176, RM539, RM454, RM494 |
| Chr.7 | RM8263, RM6449, RM418, RM3404, RM505, RM234 |
| Chr.8 | RM337, RM1376, RM1111, RM331, RM223, RM3120 |
| Chr.9 | RM257, RM278, RM3919, RM201, RM1026, RM264 |
| Chr.10 | RM5271, RM216, RM467, RM271, RM1108, RM304 |
| Chr.11 | RM268, RM1812, RM5599, RM202, RM21, RM3428 |
| Chr.12 | RM1246, RM1103, RM3226, RM270 |
